# Infiltration Profile of Regulatory T Cells in Osteoarthritis-Related Pain and Disability

**DOI:** 10.3390/biomedicines10092111

**Published:** 2022-08-29

**Authors:** Timo Albert Nees, Jiji Alexander Zhang, Hadrian Platzer, Tilman Walker, Tobias Reiner, Elena Tripel, Babak Moradi, Nils Rosshirt

**Affiliations:** 1 Department of Orthopedics, Heidelberg University Hospital, 69118 Heidelberg, Germany; 2 Clinic for Orthopedics and Trauma Surgery, University Hospital Kiel, 24105 Kiel, Germany

**Keywords:** osteoarthritis, inflammation, knee pain, T cells, regulatory T cells, lymphocytes, synovial membrane, synovial fluid, peripheral blood

## Abstract

Emerging evidence indicates that regulatory T cells (Treg) intervene in the inflammatory processes that drive osteoarthritis (OA). However, whether polarized Tregs affect clinical features of the disease in the short- or long-term, and if so, what their role in OA-related pain and functional disability really is, remains elusive. Thus, the aim of the current study was to characterize the infiltration profile of Tregs in systemic (peripheral blood) and joint-derived (synovial fluid and synovial membrane) samples from patients with knee OA in relation to OA-induced symptoms. To this end, Treg infiltration (CD4^+^CD25^+/high^ CD127^low/−^) was analyzed in matched samples of peripheral blood (PB), synovial fluid (SF) and synovial membrane (SM) from a total of 47 patients undergoing elective knee arthroplasty using flow cytometry. At the same time, knee pain and function were assessed and correlated with Treg proportions in different compartments (PB, SF, SM). Interestingly, matched-pair analysis revealed significantly higher Treg proportions in joint-derived samples than in PB, which was mainly attributed to the high Treg frequency in SF. Moreover, we found significant associations between infiltrating Tregs and OA-related symptoms which indicate that lower Treg proportions—especially in the SM—are related to increased pain and functional disability in knee OA. In conclusion, this study highlights the importance of local cellular inflammatory processes in OA pathology. Intra-articular Treg infiltration might play an important role not only in OA pathogenesis but also in the development of OA-related symptoms.

## 1. Introduction

For decades, osteoarthritis (OA) has been considered a non-inflammatory degenerative disease in which simple “wear and tear” leads to loss of articular cartilage. Recent evidence, however, demonstrates that OA development is a complex process that is driven by a plethora of inflammatory mechanisms which disrupt normal cartilage homeostasis and thus trigger joint degeneration [1,2,3,4,5,6]. Accordingly, a large proportion of OA patients present clinical signs of joint inflammation such as swelling and effusion in addition to the hallmark symptoms of OA: pain and functional disability [7,8]. Concurrently, joint inflammation seems to play a pivotal role in the development of OA-associated pain indicating important cross-talk between joint innervating nociceptive neurons and inflammatory mediators [7,8,9,10,11]. Indeed, recent studies have demonstrated that mononuclear cells (MNC) including T cells and macrophages as well as inflammatory mediators, such as nerve growth factor (NGF), interleukin 6 (IL-6) and tumor necrosis factor α (TNF α), are involved in onset and persistence of pain in both humans with OA and experimental models of arthritis [4,11,12,13,14,15,16,17,18].

In fact, a growing body of literature indicates that patients suffering from chronic pain conditions present a different phenotypic profile of circulating T cells when compared to controls [19,20]. Moreover, T cells are involved in the onset but also the resolution of pain which underpins their supposed role in the transition from acute to chronic pain. In preclinical models, the different effects of T cells during pain development depend on the type of pain model, gender and, most importantly, on the subset of T cells. T-helper cells (Th), especially, are characterized by the expression of the surface marker cluster of differentiation 4 (CD4), including functionally different Th subsets such as Th1, Th2, Th17, and regulatory T cells (Tregs) have been reported to mediate pain development [19]. Whereas Th1 and Th17 are thought to increase pain sensitivity by the production of pro-inflammatory cytokines, Th2 and Treg subsets potentially ameliorate pain by releasing anti-inflammatory mediators (e.g., IL-10) and endogenous opioids. Thus, influencing Th-cell responses in OA could be a promising therapeutic strategy to attenuate joint pain [19,20].

In recent years, Tregs have been increasingly studied since they are important regulators of immune responses in inflammatory and autoimmune diseases [20]. Their main function is to control and suppress the activity of innate and adaptive immune cells including other T-cell subsets. Indeed, we and others have demonstrated that decreased Treg responses may be involved in the pathogenesis of OA and RA as the subsequent reduction of anti-inflammatory IL-10 release results in an exacerbation of inflammatory processes that drive arthritis progression [20,21]. For example, it was shown that Treg proportions are reduced at the site of synovial inflammation in RA patients [22] and that OA patients have lower numbers of Tregs in the peripheral blood when compared to age-matched healthy controls [23]. In addition, RA and OA patients share profound commonalities regarding the infiltration profile of Tregs in peripheral blood (PB), synovial fluid (SF) and synovial membrane (SM), with greater accumulation of Tregs in the affected joints (SF and SM) [21].

Interestingly, emerging evidence from preclinical studies support a vital role for Tregs in pain processing. It was suggested that Treg infiltration promotes pain recovery in animal models of peripheral and central neuropathic pain as well as experimental arthritis [24]. In fact, depletion of Tregs prolonged mechanical hypersensitivity after peripheral nerve injury [25,26,27,28], whereas both Treg infiltration [28] and adoptive Treg cell transfer attenuated neuropathic pain [29].

Nevertheless, the role of Tregs in OA-related pain—which is predominantly inflammatory—remains elusive. Thus, our study aimed to (i) map the infiltration pattern of Tregs in PB, SF and SM samples from knee OA patients and to (ii) assess potential relations between the compartmental Treg profile and clinical symptoms, including OA-induced pain and functional disability.

## 2. Materials and Methods

### 2.1. Patient Enrollment

OA was defined according to the American College of Rheumatology criteria and was classified as unicompartmental (UC) or bicompartmental (BC) OA based on plain radiographs. UC OA patients were scheduled for unicompartmental (UKA) and those with BC OA for total knee arthroplasty (TKA). None of the patients had a history of underlying inflammatory pathology, intake of disease-modifying anti-rheumatic drugs (DMARD) or intra-articular injection of corticosteroids or hyaluronic acid. Systemic inflammatory parameters (CRP and WBC) were within the physiological range at the time of surgery. The study was conducted in accordance with the local ethics committee of the Medical Faculty at Heidelberg University and the declaration of Helsinki. It was approved by the institutional review board of the Medical Faculty Heidelberg (S333/2007). Informed consent of all patients was obtained prior to study enrollment.

### 2.2. Clinical Assessment

Radiographic OA severity was graded according to the Kellgren and Lawrence (K&L) scoring system (0–IV) [30] using anteroposterior radiographs of the symptomatic knees. Prior to surgery, knee pain and function were assessed using the 11-point (0–10) numerical rating scale (NRS; 0 = no pain; 10 = worst pain), the 12-item self-administered Oxford Knee Score (OKS-12) [31], the American Knee Society Score (AKSS) [32] and the Hannover Functional Questionnaire of Functional Disability Caused by OA (FFbH-OA).

### 2.3. Sample Collection

PB, SF and SM samples were collected at the time of surgery as previously described [33]. Prior to arthrotomy, SF was removed by needle aspiration and stored in sterile tubes until further processing. SM samples were harvested from the suprapatellar pouch after arthrotomy. Concurrently, heparinized PB samples were collected. For overall analyses, *n* = 43 PB, *n* = 17 SF and *n* = 38 SM samples were available. Dry taps (punctio sicca) and excessive contamination of SF samples with blood during needle aspiration account for the lower number of SF samples when compared to the number of SM samples.

### 2.4. Sample Processing

PB, SF and SM samples were prepared directly after harvesting for further flow cytometry analysis as previously described [33]. In brief, SF samples were incubated with bovine testicular hyaluronidase (10 mg/mL; Sigma-Aldrich, St. Louis, MO, USA) for 30 min at 37 °C and washed twice with phosphate-buffered saline (PBS). SM samples were washed twice with PBS and minced finely with sterilized scissors before being digested with collagenase B (1 mg/mL; Roche Diagnostics, Rotkreuz, Switzerland) and bovine testicular hyaluronidase (2 mg/mL) in RPMI-1640 culture medium supplemented with penicillin–streptomycin (10 µg/mL; Invitrogen, Carlsbad, CA, USA) and 5% fetal calf serum (FCS, Biochrom AG, Berlin, Germany) at 37 °C for 2 h. After digestion the SM cell suspension was consecutively filtered through 100 µm (BD Biosciences, San Jose, CA, USA) and 40 µm pore-size cell strainer (EMD Millipore, Burlington, MA, USA) to remove any undigested tissue. Then, the filtered SM cell suspension was washed twice with PBS. To isolate mononuclear cells (MNC) from the filtered SM and SF cell suspensions as well as the heparinized PB samples, Ficoll–Paque™ PLUS (GE Healthcare, Chicago, IL, USA) density gradient centrifugation was used according to the manufacturer’s instructions. Subsequently, T cells (CD3^+^) were isolated from PB-, SF- and SM-MNC using CD3 magnetic activated cell sorting (MACS) bead separation (Miltenyi Biotec, Bergisch Gladbach, Germany).

### 2.5. Flow Cytometry Analyses of Cell Surface Markers

Multicolor flow cytometry was used to analyze Treg infiltration in PB, SF and SM samples. Tregs are characterized by the expression of the following distinct surface markers: CD4^+^CD25^+/high^ CD127^low/−^. Thus, to identify the Treg subset, isolated T cells were stained for these Treg specific surface markers. To this end, CD3^+^ MACS isolated T cells from PB, SF and SM were washed twice in staining buffer, blocked with FcR blocking reagent (Miltenyi Biotec) and then stained for 30 min at 4 °C with fluorescein isothiocyanate (FITC)-labeled monoclonal antibody (mAb) against CD4 (clone RPA-T4; BD Biosciences), phycoerythrin (PE)-labeled mAb against CD25 (clone M-A251; BD Biosciences) and peridinin–chlorophyll–cyanin 5.5 (PerCPCy5.5)-labeled mAb against CD127 (clone RDR5; eBioscience, San Diego, CA, USA). After staining, cells were washed again and taken into a final volume of 200 µL MACS staining buffer. Before flow cytometric detection, 0·5 μg/mL 7-aminoactinomycin D (7 AAD) (eBioscience) was added to the cell suspensions to exclude cell debris and dead cells. Flow analysis was performed using a MACSQuant Analyzer (Miltenyi Biotec, Germany). Data analysis was carried out using FlowJo™ version 10.8.1 (Ashland, OR: Becton, Dickinson and Company, Franklin Lakes, NJ, USA). The cut-off for all cell surface markers was defined based on fluorescence minus one (FMO) controls, as described previously [21].

### 2.6. Gating Strategy and Definition of the Treg Population

**Figure 1:**
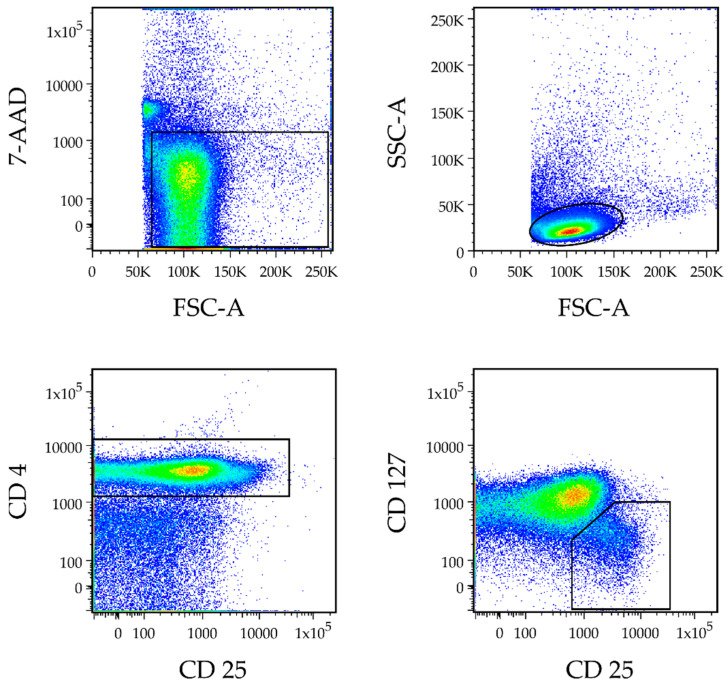
Gating strategy for regulatory T cells (Treg). Representative dot plots of the gating strategy for Tregs are shown. Based on forward (FSC-A) and side scatter (SSC-A) profiles, cells were gated for lymphocytes and further for CD4 expression, and 7-aminoactinomycin D (7-AAD) was used to exclude cell debris and dead cells. By labeling the cell surface markers CD25 and CD127, the Treg population was identified as CD4^+^CD25^+/high^CD127^−/low^. The CD4^+^ cells with the highest level of CD25 staining were defined as CD4^+^CD25^high^ cells. The CD4^+^ CD25^+/high^CD127^−/low^ Treg population was distinct and clearly separable from other cells. CD = cluster of differentiation.

Based on forward and side scatter profiles, cells were gated for lymphocytes and further for CD4 expression. By labeling the cell surface markers CD25 and CD127, the Treg population was identified as CD4^+^CD25^+/high^ CD127^low/−^ Tregs. The cutoff for all cell surface markers was established based on FMO controls. The CD4^+^ cells with the highest level of CD25 staining were defined as CD4^+^CD25^high^ cells. The CD4^+^CD25^+/high^ CD127^low/−^ Treg population was distinct and clearly separable from other cells as previously described [34]. The gating strategy is illustrated in Figure 1.

### 2.7. Statistical Analyses

Descriptive statistics of demographic and clinical parameters are expressed as mean ± standard deviation (SD) and range. Descriptive data of the flow cytometry analyses are presented as median and interquartile range (IQR) or as mean ± standard error of the mean (SEM), including the 95% confidence interval (CI). To reveal differences in Treg cell distribution between distinct samples (PB, SF, SM) analysis of variance was performed. Due to the predominantly non-parametric distribution of Treg cells, Kruskal–Wallis test followed by Dunn’s multiple comparison test were used to determine overall group differences. For matched-paired analysis Wilcoxon matched-pairs signed rank test was used. Spearman’s rank correlation coefficient was performed to examine correlations between Treg proportions and clinical parameters (K&L score, NRS, OKS-12, AKSS, FFbH-OA). *p*-Values < 0.05 were considered statistically significant. Prism version 9.0 (GraphPad Software Inc., La Jolla, CA, USA) was used for statistical analysis.

## 3. Results

### 3.1. Clinical Characteristics of the Study Population

Table 1 summarizes the clinical characteristics of the study population.

In brief, a total of 46 patients were included in this study. The majority of the patients were female (73.9% vs. 26.1% male) and received total knee replacement (65.2% vs. UKA 34.8%). Age ranged from 47 to 83 years and was on average 67.7 ± 8.7 years. With a mean body mass index (BMI, ± SD) of 29.8 ± 6.2 kg/m^2^, the study population can be considered overweight. Almost two-thirds of the patients (60.9%) had K&L score IV, whereas 17.4% and 21.7% were graded K&L II and III, respectively. Mean knee pain intensity (±SD) on the NRS was rated 7.2 (±2.1). On average patients scored 33.9 (±9.1) points in the OKS-12 questionnaire. Mean AKSS knee and functional scores were 42.7 (±15.4) and 56.3 (±21.6), respectively. Laboratory results indicating systemic inflammation were within the physiological range.

### 3.2. Treg Profile in OA Joints (SF, SM) and PB

To characterize the infiltration pattern of Tregs in different compartments from knee OA patients, flow cytometry data of matching PB, SF and SM samples were analyzed. Table 2 summarizes the overall results from the multicolor flow cytometry.

In brief, CD4^+^ T cells (mean ± SEM) accounted for 77.35 ± 2.23% (CI: 72.84–81.85), 44.57 ± 3.35% (CI: 37.62–51.52) and 75.52 ± 1.29% (CI: 72.90–78.14) of MACS isolated T lymphocytes in PB, SF and SM, respectively. The highest CD4^+^ cell concentrations (mean ± SEM) were measured in PB (10,660 ± 817.1 cells/mL, CI: 9011–12,309) and the lowest in SF (742.9 ± 288.2 cells/mL, CI: 145.3–1341).

**Figure 2:**
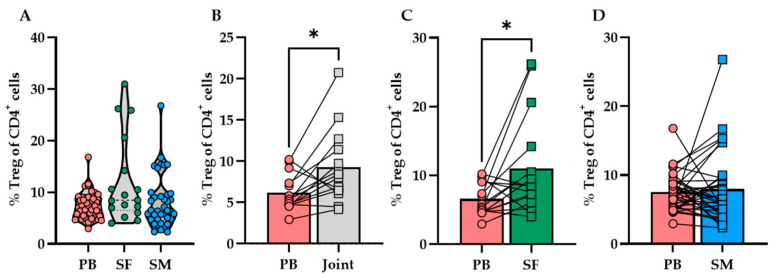
Regulatory T-cell (Treg) infiltration profile in OA joints and peripheral blood. (**A**) Overview of the mean Treg proportions (% Treg of CD4^+^ cells) in peripheral blood (PB), synovial fluid (SF) and synovial membrane (SM). (**B**–**D**) Matched-pairs analysis comparing mean Treg proportions in PB with the mean Treg proportions in (**B**) joint-derived samples (SF and SM), (**C**) SF and (**C**) SM alone. (**A**) Comparing the mean Treg proportions (% Treg of CD4^+^ cells) in peripheral blood (PB), synovial fluid (SF) and synovial membrane (SM) did not show significant differences between tissue types (Kruskal–Wallis test, *p* = 0.1636). (**B**–**D**) However, matched-pairs analysis demonstrated (**B**) significantly higher Treg proportions in joint-derived samples (SF and SM) than in PB (Wilcoxon matched-pairs signed rank test, *p* = 0.0203, n = 14), which can be mainly attributed to (**C**) high infiltration rates of Tregs in SF (Wilcoxon matched-pairs signed rank test, *p* = 0.0250, n = 16) rather than (**D**) the Treg proportions in SM (Wilcoxon matched-pairs signed rank test, *p* = 0.8907, n = 35). *p*-Values < 0.05 were considered statistically significant and are indicated with asterisks: * *p* < 0.05.

Accordingly, Treg concentrations differed significantly between compartments (PB/SF/SM). The lowest Treg concentrations (mean ± SEM) were measured in SF (135.8 ± 71.88 cells/mL, CI: 17.42–289). In PB and SM, Tregs were higher concentrated as indicated by 773.5 ± 65.64 cells/mL (CI: 641.1–906) and 853.5 ± 444.8 cells/g (CI: 49.54–1757), respectively (PB vs. SF: **** *p* <0.0001, PB vs. SM: *** *p* = 0.0005, SF vs. SM: * *p* = 0.03; Kruskal–Wallis test followed by Dunn’s multiple comparisons test). In contrast, the highest proportion of infiltrating Tregs (12.17 ± 2.05% of CD4^+^ T cells) was found in SF. Although there were statistically no significant differences between sample localizations (PB, SF, SM) when comparing the mean Treg proportions (% of CD4^+^ T cells) of all samples, matched-pair analysis revealed significantly greater infiltration of Tregs in joint compartments (SF and SM) than in PB, which is most likely driven by high Treg infiltration rates in SF (see Figure 2). Thus, the percentage rates of Tregs in SF were significantly higher than in PB (Wilcoxon matched-pairs signed rank test, *p* = 0.0250, n = 16), whereas there was no difference between Treg proportions in PB and SM.

### 3.3. Obesity Is Associated with Decreased Treg Infiltration in SM

**Figure 3:**
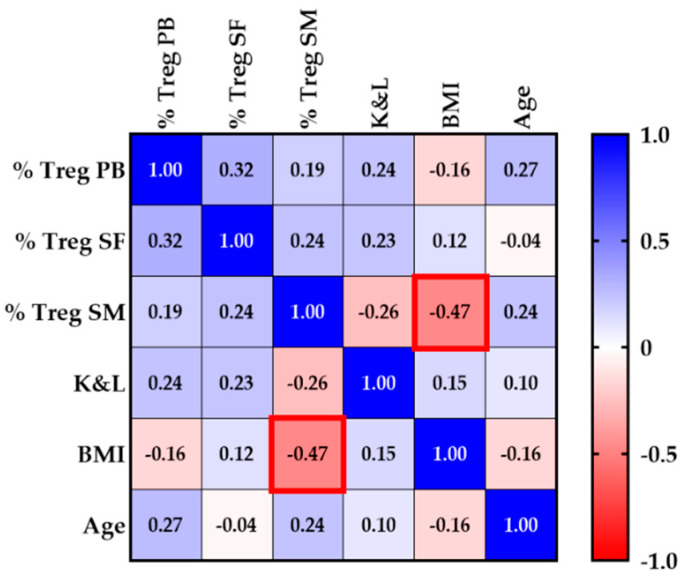
Heatmap of the correlation matrix screening for relevant associations between Treg proportions (% Treg) in peripheral blood (PB), synovial fluid (SF) and synovial membrane (SM) and OA-severity (K&L score), age and body mass index (BMI). A significant negative correlation was found between Treg proportions in SM and the BMI (bold red square; *p* = 0.003, r_s_ = −0.47). Values displayed in the squares of the correlation matrix represent the Spearman’s rank correlation coefficient (r_s_) for every pair of data set. Bold red squares highlight significant correlations (*p*-value < 0.05). K&L score = Kellgren and Lawrence score.

First, we examined whether Treg infiltration rates are associated with age, BMI or OA severity (K&L score) as displayed in the correlation matrix (see Figure 3). Treg proportions in all tissues (PB, SF, SM) were not related to age or OA-grade. However, we found a moderate negative correlation between Treg proportions in SM and the weight of knee OA patients (*p* = 0.003, r_s_ = −0.47), indicating that patients with higher BMI scores present lower synovial Treg infiltration rates (see Figure 3).

### 3.4. Functional Disability Is Associated with Decreased Treg Infiltration in SM

**Figure 4:**
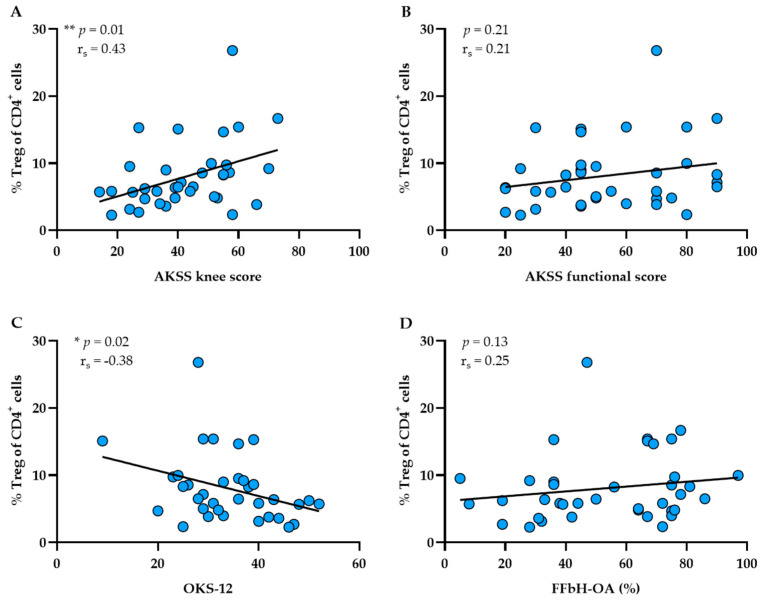
Correlation analyses between synovial membrane (SM) infiltration of Tregs and functional parameters of knee OA patients. Spearman’s rank correlation coefficient (r_s_) revealed that Treg proportions (% Treg) significantly correlated with knee joint function. Higher percentage rates of Tregs in SM were associated with better scores in the (**A**) AKKS knee and (**C**) OKS-12. No significant associations were found between Treg proportions and AKSS functional (**B**) and FFbH-OA scores (**D**). *p*-Values < 0.05 were considered statistically significant and are indicated with asterisks: * *p* < 0.05, ** *p* < 0.01. AKSS = American Knee Society Score; FFbH-OA = Hannover Functional Questionnaire of Functional Disability Caused by OA; OKS-12 = Oxford Knee Score.

To examine the influence of compartment-specific Treg infiltration on OA-related knee function, Treg proportions in PB, SF and SM were correlated with clinical assessment scores (AKSS, OKS-12 and FFbH-OA). Interestingly, we observed no significant correlations between Treg proportions in PB and SF and knee function (see Supplementary Figure S1). However, Treg infiltration rates in SM significantly correlated with AKSS knee (*p* = 0.01, r_s_ = 0.43) and OKS-12 scores (*p* = 0.02, r_s_ = −0.38), which suggests that OA patients with worse knee function present decreasing synovial Treg infiltration (see Figure 4A–D).

### 3.5. Knee Pain Is Associated with Decreased Treg Infiltration in SM and PB

**Figure 5:**
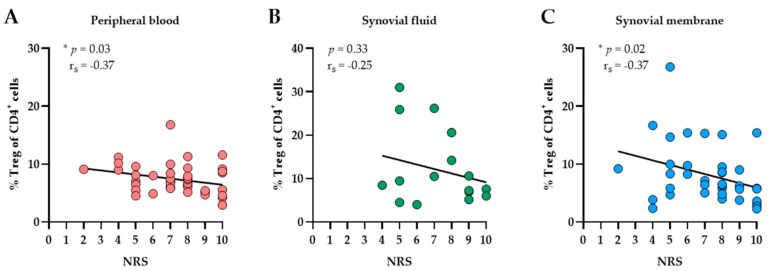
Correlation analyses between tissue-specific infiltration of Tregs and pain of knee OA patients. Spearman’s rank correlation coefficient (r_s_) revealed that Treg proportions (% Treg) in (**A**) peripheral blood and (**C**) synovial membrane (SM) significantly correlated with knee pain intensity measured on a numerical rating scale (NRS). Higher pain levels were associated with decreasing Treg proportions in PB and SM. (**B**) In synovial fluid, no associations between Treg proportions and pain scores were observed. *p*-Values < 0.05 were considered statistically significant and are indicated with asterisks: * *p* < 0.05.

We next examined associations between compartment-specific Treg infiltration and OA-related knee pain. Correlation analyses (see Figure 5) revealed that increasing proportions of Tregs in PB are associated with lower pain levels (*p* = 0.03, r_s_ = −0.34). Similarly, we found a significant negative correlation between Treg infiltration in SM and knee pain ratings (*p* = 0.02, r_s_ = −0.37). These results indicate that knee OA patients with lower proportions of infiltrating Tregs in both PB and SM have higher levels of knee pain.

## 4. Discussion

In summary, the current study analyzed the infiltration profile of Tregs in different compartments of knee OA-patients and screened for potential associations between Treg infiltration and OA-related symptoms. In line with previous results, we could demonstrate that Treg proportions are enriched in joint-derived samples (SF and SM) when compared to the percentage of Tregs in PB. This is mainly attributed to high Treg frequencies in SF. Moreover, we found significant coherences between infiltrating Tregs and OA-related symptoms which indicate that lower Treg proportions—especially in SM—are related to increased pain and functional disability in knee OA. This is in line with recent data by our group, analyzing mainly Kellgren and Lawrence stage IV OA patients [33]. To our knowledge this is the first study to evaluate Treg infiltration in relation to clinical symptoms of knee OA patients.

Recent evidence from both clinical and preclinical studies indicates that Tregs play an important role in the complex inflammatory cascade that triggers OA. They modulate the secretion of anti-inflammatory cytokines such as IL-10 [20,22,35]. Thus, reduced Treg responses in inflammatory diseases are thought to shift the balance between pro- and anti-inflammatory processes towards inflammation. This is proven to drive disease progression in animal models for antigen induced arthritis (AIA) and collagen-induced arthritis (CIA) [36,37]. Similarly, it has been reported that OA patients have fewer Treg cells in PB when compared to age-matched healthy controls [23]. Here, we also found reduced Treg proportions in PB when compared to joint-derived samples. However, conclusions regarding healthy controls cannot be drawn since we did not harvest control samples from healthy individuals due to ethical reasons.

In line with our previous findings, the percentage of Tregs was highest in SF samples followed by SM and PB. In SF, Tregs accounted for ~12% of CD4^+^ T cells. Differences between Treg proportions in tissue types could be explained by the physiology of Treg compartmentalization which has been reported to be organ- and tissue-specific. The underlying mechanisms might include compartment-specific Treg trafficking and retention. In line with this, we have previously shown that in synovial samples of end-stage OA knee joints, a pro inflammatory Th1 polarization is predominant [33]. Therefore, we hypothesize that the high Treg proportions observed in OA-joints are present to counterbalance the pro inflammatory milieu with their anti-inflammatory properties. Mechanistically, Tregs develop their suppressive activity in a contact-dependent manner [38], thereby emphasizing the importance of Treg infiltration into the joint (SF and SM). Furthermore, the importance of SM-infiltrating Tregs is underlined by the fact that soluble factors alone are unable to exhibit immunosuppressive activity [21,39,40,41]. Once activated in the SM, Tregs can release the anti-inflammatory IL-10 to suppress Th1 and/or cytotoxic T-cell activation [42,43,44]. Interestingly, IL-10 secretion from PB Tregs of OA-patients was found to be decreased despite elevated Treg frequencies when compared to non-OA controls [42]. Decreased IL-10 secretion from Tregs was accompanied by reduced Tim-3 expression on Tregs which is a marker of T-cell exhaustion. This suggests that decreased IL-10 release by Tregs might be related to a suppressed capacity of Tim-3^+^ Tregs to produce IL10 in OA [44]. Taken together, the high Treg proportions in joint-derived samples in combination with the fact that patients were admitted for arthroplasty indicate that the counterbalancing capacity of Tregs to restore inflammatory homeostasis is limited.

Nevertheless, Treg proportions in SM seem to be related to the degree of OA-induced symptoms. Both knee pain and functional disability were significantly associated with decreased Treg proportions in SM indicating important cross-talk with joint innervating nociceptive neurons. The majority of knee joint nociceptors are located in deep somatic tissue including the joint capsule, insertion of tendons, ligaments, subchondral bone and periost [1,45,46]. The synovium, especially, is one of the most densely innervated intra-articular structures of the knee joint, and the degree of synovitis in MRI studies highly correlates with knee pain [7,8,9]. Therefore, Tregs infiltrating the SM have a high potential to mediate nociceptor activation. Their role in pain processing has been studied in several neuropathic pain models, including partial sciatic nerve ligation (PSNL), chronic constriction injury (CCI), diabetic painful neuropathy (DPN) and chemotherapy-induced peripheral neuropathy (CIPN) [19]. After experimental Treg depletion, mechanical allodynia was significantly enhanced in response to CCI [25] and CIPN was reduced following intrathecal injection of Tregs [28]. Despite these promising results, the mechanisms underlying the pain-alleviating effect of Tregs remains elusive. Most likely, it is also mediated through their capacity to shift the milieu to a more anti-inflammatory environment by IL-10 secretion [19]. In fact, many pain ameliorating Treg effects can be reproduced by IL-10 application and are absent in mice lacking IL-10 [47,48,49,50]. Therefore, IL-10 seems to be a key factor in Treg-mediated pain attenuation. Nevertheless, it is unclear if Tregs produce sufficient IL-10 by themselves to resolve pain or if they induce IL-10 secretion in other cell types [48,50,51]. In fact, IL-13 released by Tregs induces IL-10 secretion in IL13R^+^ macrophages in models of acute systemic inflammation [51]. Although the exact mechanisms remain unclear, the growing body of literature supports a pain-alleviating role of Tregs.

Of note, the Treg infiltration data might be influenced by sociodemographic and clinical parameters such as age, OA-severity and BMI. Indeed, we found that patients with higher BMI scores presented decreased Treg proportions in the SM. This is in line with previous reports, demonstrating that circulating Tregs are reduced in obese patients when compared to patients with normal body weight [52]. Furthermore, compartment-specific reduction of Tregs in adipose tissue of both obese humans and mice has been reported [53]. Here, we also found a significant compartment-specific association between reduced Tregs in SM and increasing body weight. Due to the limited and different numbers of matching data per variable adequate adjustments for confounding factors were not performed. Although OA severity ranged from K&L scores II to IV, all patients suffered from clinically advanced OA and therefore underwent knee replacement surgery. Thus, the reported Treg profile represents the infiltration status of patients requiring surgery due to relevant OA-induced symptoms. However, generalization of our findings needs to be done with caution as early and intermediate OA stages present a different Treg infiltration patterns, as we showed in a previous work by our group [54]. Especially the relation of pro- and anti-inflammatory T cells seems to shift during disease progression. Furthermore, data interpretation might also be affected by the method of Treg identification due to the lack of one specific Treg marker. Here, we used the surface markers CD4, CD125 and CD127 to detect viable Tregs (CD4^+^CD25^+/high^ CD127^low/-^) after isolation of CD3^+^ T lymphocytes (MACS). In general, the specificity of Treg detection has been improved with labeling of FoxP3—a transcription factor which is required for Treg development. However, due to its intracellular location FoxP3 does not allow separation of viable cells [21,55]. Thus, we used CD127 as an additional surface marker, in combination with CD25, which has been shown to facilitate consistent quantitative identification of viable Tregs (CD4^+^CD25^+/high^ CD127^low/−^), which are highly positive for FoxP3 [34,40,56]. Different techniques of identifying Tregs might partly explain contradicting results of studies assessing Treg frequencies in tissue samples [21,39,41,54,55,56,57,58,59,60]. Moreover, variability across patients might affect the interpretation of the results but was not tested in detail.

In conclusion, our study provides a solid foundation for further experimental and clinical studies to untangle the role of Tregs in OA-related symptoms by identifying specific Treg profiles and their potential associations with clinical characteristics.

## Figures and Tables

**Table 1:** Study population.

Total Study Population	
Number of Patients (N=)	46
Gender	
Female, *n* (%)	34 (73.9%)
Male, *n* (%)	12 (26.1%)
Age, *years*	67.7 ± 8.7 (47–83)
BMI, *kg/m^2^*	29.8 ± 6.3 (20.3–50.1)
Laboratory results	
CRP, *mg/L*	3.8 ± 0.6
WBC, *cells/nL*	6.9 ± 0.24
ESR, *mm/h*	16.0 ± 1.7
Knee replacement	
UKA, *n* (%)	16 (34.8%)
TKA, *n* (%)	30 (65.2%)
K&L score, *n* (%)	
I	0 (0%)
II	8 (17.4%)
III	10 (21.7%)
IV	28 (60.9%)
Knee pain, *NRS pt*.	7.2 ± 2.1 (2.0–10.0)
OKS-12, *pt.*	33.9 ± 9.1 (9.0–52.0)
AKSS	
Knee score, *pt*.	42.7 ± 15.4 (14.0–73.0)
Functional score, *pt.*	56.3 ± 21.6 (20.0–90.0)
FFbH-OA (%)	54.0 ± 23.1 (5.0–97.0)

Clinical characteristics and assessment scores are presented as mean ± SD (range). Laboratory results are displayed as mean ± SEM. BMI = body mass index, CRP = C-reactive protein, WBC = white blood cells, ESR = erythrocyte sedimentation rate, UKA = unicompartmental knee arthroplasty, TKA = total knee arthroplasty, K&L score = Kellgren and Lawrence score, NRS = numerical rating scale, OKS-12 = Oxford Knee Score, AKSS = American Knee Society Score, FFbH-OA = Hannover Functional Questionnaire of Functional Disability Caused by OA.

**Table 2:** T cell infiltration in peripheral blood, synovial fluid and synovial membrane.

	PB	SF	SM
**Sample volume/weight** (PB, SF: *mL*, SM: *g*)	8.3 (8.0–8.5)	6.0 (3.25–17.25)	2.89 (2.18–3.12)
**CD3^+^ MACS isolated T lymphocytes**			
Cell count	113,122 (95,377–131,436)	1965 (269–16,983)	9477 (3425–32,652)
**CD4^+^ T cells**			
Cell count	89,706 (72,045–111,883)	1294 (88–5129)	6972 (2536–25,707)
Cell concentration (PB, SF: *cells/mL*, SM: *cells/g*)	10,764 (7007–13,510)	178.2 (8.01–740.5)	2471 (873.1–8952)
% of T lymphocytes	80.20 (74.20–86.70)	45.30 (32.10–52.40)	78.40 (69.68–81.70)
**Tregs (CD4^+^CD25^+/high^CD127^low/−^)**			
Cell count	6200 (4006–8724)	170 (44–1191)	557 (242–1776)
Cell concentration (PB, SF: cells/mL, SM: cells/g)	721.3 (448.6–1135)	46.32 (7.92–142.8)	169.1 (76.37–609.7)
% of CD4^+^ T cells	7.03 (5.40–9.06)	8.47 (6.52–17.40)	6.43 (4.80–9.60)

The distribution of T cells in matching peripheral blood (PB), synovial fluid (SF) and synovial membrane (SM) samples of knee OA patients was assessed using flow cytometry. For CD3^+^ magnetic cell separation (MACS), isolated T lymphocyte median cell counts and interquartile ranges (IQR) are presented. For both the CD4^+^ and Treg subpopulation cell counts, concentration levels (cells/sample volume (mL) or weight (g)) and percentage rates (%) are shown as medians (IQR). CD = cluster of differentiation.

## Data Availability

All data are available in the manuscript or supplemental information. Any additional information (such as raw data) is available from the corresponding author upon reasonable request.

## Supplementary Materials

The following supporting information can be downloaded at: https://www.mdpi.com/article/10.3390/biomedicines10092111/s1, Figure S1: Correlation analyses between Tregs in peripheral blood, synovial fluid and functional parameters of knee OA patients.
