# Supplementary material for: Infiltration Profile of Regulatory T Cells in Osteoarthritis-Related Pain and Disability
Source: Biomedicines. 2022 Aug 29;10(9):2111. doi: 10.3390/biomedicines10092111 (PMC9495462; doi:10.3390/biomedicines10092111)
Supplement: Supplementary file 1 [file biomedicines-10-02111-s001.zip › biomedicines-1826977-supplementary.pdf]

Supplementary Material:

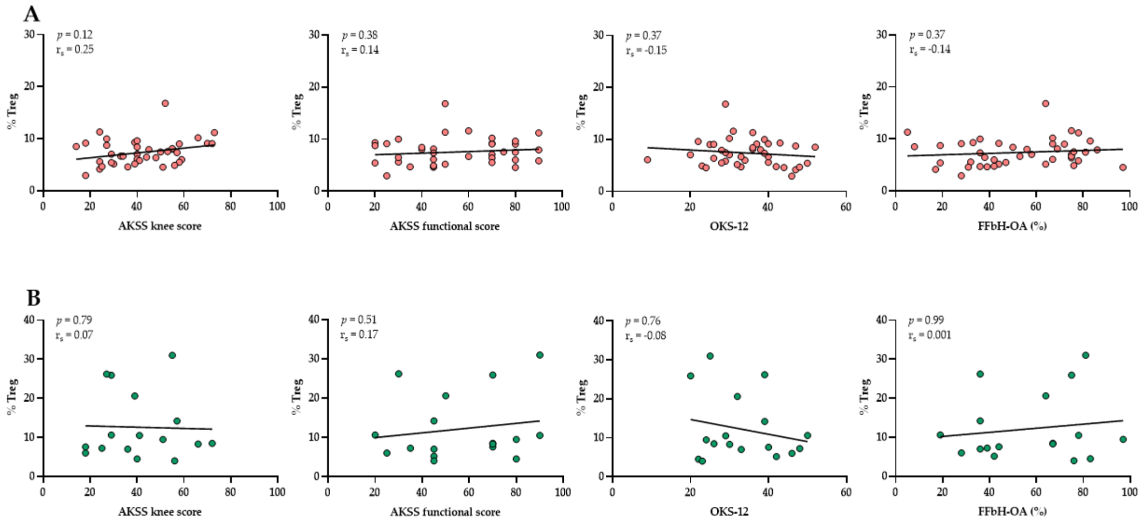

Figure S1. Correlation analyses between Tregs in (**A**) peripheral blood (PB, red scatter dot plots) and (**B**) synovial fluid (SF, green scatter dot plots) and functional parameters of knee OA patients. Spearman's rank correlation coefficient (r_s) revealed that Treg proportions (% Treg) in PB and SF do not correlate with functional parameters (AKKS, OKS-12, FFbH-OA %). p-Values < 0.05 were considered statistically. AKSS = American Knee Society score; FFbH-OA = Hannover Functional Questionnaire of functional disability caused by OA; OKS-12 = Oxford Knee Score.
